# Subcutaneous Thrombotic Vasculopathy with Features of Leukocytoclastic Vasculitis Following Intravenous Injection of Crushed Oxycodone and Methylphenidate Tablets: A Case Report with Literature Review

**DOI:** 10.3390/jcm15114044

**Published:** 2026-05-23

**Authors:** Nina Łabędź, Maksymilian Markwitz, Paweł Głuszak, Monika Bowszyc-Dmochowska, Marian Dmochowski, Adriana Polańska, Aleksandra Dańczak-Pazdrowska

**Affiliations:** 1 Department of Dermatology, Poznan University of Medical Sciences, 60-355 Poznan, Poland; 2 Doctoral School, Poznan University of Medical Sciences, 60-812 Poznan, Poland; 3 Cutaneous Histopathology and Immunopathology Section, Department of Dermatology, Poznan University of Medical Sciences, 60-355 Poznan, Poland; 4 Autoimmune Blistering Dermatoses Section, Department of Dermatology, Poznan University of Medical Sciences, 60-355 Poznan, Poland

**Keywords:** subcutaneous thrombotic vasculopathy, leukocytoclastic vasculitis, intravenous drug use, oxycodone, cutaneous microvascular thrombosis, case report

## Abstract

Subcutaneous thrombotic vasculopathy (STV) is a rare, non-inflammatory occlusive disorder of the cutaneous microvasculature that predominantly involves the subcutaneous tissue and may closely mimic inflammatory vasculitis. We describe a case of STV with overlapping features of leukocytoclastic vasculitis (LCV) in a 23-year-old woman presenting with rapidly progressive, painful purpuric skin lesions. The patient had a history of polysubstance use disorder and reported intravenous injection of crushed oral oxycodone and methylphenidate tablets. Histopathological examination of a deep skin biopsy revealed fibrin-rich thrombi occluding small vessels of the dermis and subcutaneous tissue. Fine granular IgA deposits in the walls of numerous superficial dermal blood vessels shown using direct immunofluorescence suggested LCV. Overall, the findings supported a mixed thrombotic-inflammatory vasculopathy with predominant features of STV. This case highlights the diagnostic complexity of STV and may suggest intravenous injection of crushed oral medications as a potential trigger through particle-induced microvascular obstruction and secondary thrombosis. In addition, we conducted a literature review indicating that STV remains a rare and likely underrecognized entity, with only a limited number of reported cases.

## 1. Introduction

Subcutaneous thrombotic vasculopathy (STV) is a rare, non-inflammatory occlusive vasculopathy of the skin. It belongs to the broader group of occlusive thrombotic vasculopathies, a heterogeneous category of vascular disorders that frequently mimic vasculitis both clinically and histopathologically. STV is characterized by extensive microvascular thrombosis predominantly affecting small vessels and capillaries of the subcutaneous tissue. However, unlike inflammatory vasculitides, STV is defined by the absence of vessel wall inflammation, fibrinoid necrosis, and immune complex deposition [1]. However, secondary inflammatory changes and overlapping vasculitic features may occasionally occur, particularly in evolving lesions [1].

STV was first described as a distinct clinicopathologic disorder by Zembowicz et al. in 2011 [1], based on a series of patients presenting with painful skin necrosis and histologic findings of diffuse thrombosis confined to the subcutaneous microvasculature without vascular calcification, a feature that distinguishes STV from classic calciphylaxis but does not exclude a shared pathogenetic background. Calciphylaxis is considered a non-inflammatory occlusive vasculopathy in which microvascular thrombosis and ischemic injury are early and central events, whereas vascular calcification may be focal, patchy, or absent in early disease [1,2]. Consequently, STV has been hypothesized to represent either a distinct thrombotic vasculopathy or an early, non-calcified stage of calciphylaxis (calciphylaxis sine calcifications) rather than vasculitis [1].

Clinically, STV typically presents with painful retiform purpura, livedo racemosa, subcutaneous nodules, and rapidly progressive skin necrosis [1]. The lesions typically involve regions rich in subcutaneous fat, such as the thighs, buttocks, lower abdomen, and proximal lower extremities [1]. Systemic symptoms are often absent or mild, and laboratory findings are usually nonspecific [1].

Histopathologically, STV is characterized by fibrin-rich thrombi occluding small vessels of the dermis and, most prominently, the subcutaneous adipose tissue. The vessel walls are preserved, without inflammatory infiltrates or fibrinoid necrosis [1]. Direct immunofluorescence is negative or shows only scant, nonspecific deposits [1].

STV remains a rare condition. Since its original description, only a limited number of well-documented cases and small case series have been published. In this report, we describe a case of STV with overlapping features of LCV following intravenous injection of crushed oral medications and provide a review of the literature. To the best of our knowledge, this may represent one of the first reported cases associated with intravenous injection of crushed oxycodone and methylphenidate tablets in a young adult woman.

## 2. Case Report

### 2.1. Patient History

A 23-year-old woman was admitted to the Department of Dermatology due to rapidly progressive purpuric skin lesions that had developed over several days. They were localized on the face (particularly earlobes), right upper extremity, and both thighs, accompanied by fever up to 39 °C. Prior to dermatologic admission, she had been hospitalized in the Department of Cardiology for diagnostic evaluation. Infective endocarditis was excluded, and repeated blood cultures were negative. Computed tomography angiography of the head, chest, and abdomen revealed no vascular abnormalities. Doppler ultrasonography of the right upper limb demonstrated thrombosis of superficial veins, and treatment with low-molecular-weight heparin (enoxaparin) was initiated, with a recommendation for continued outpatient anticoagulation. Broad-spectrum intravenous antibiotic therapy was also initiated, resulting in decreased inflammatory markers and improved the patient’s general condition. After discharge from the Cardiology Department, the patient discontinued anticoagulation on her own. Shortly thereafter, she noted the abrupt appearance of new, painful purpuric lesions on both thighs. Her medical history was notable for borderline personality disorder and polysubstance use disorder. She admitted to intravenous use of crushed oxycodone (oxycodone hydrochloride) and methylphenidate. However, she was unable to specify the exact timing of the last injection. Chronic medications included quetiapine, pregabalin, chlorprothixene, lamotrigine, propranolol, risperidone, and trazodone. No history of inflammatory bowel disease was reported.

### 2.2. Physical Examination and Diagnostic Assessment

**Figure 1:**
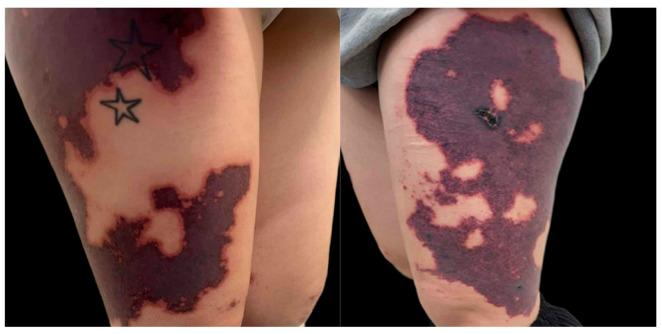
Clinical presentation at admission. Confluent, painful, violaceous purpuric patches involving both thighs, consistent with retiform purpura.

On dermatologic examination at admission, confluent, painful, violaceous purpuric patches consistent with retiform purpura were present on both thighs within the lesions, tens of hemorrhagic bullae and erosions were visible (Figure 1).

Laboratory investigations revealed markedly elevated C-reactive protein (CRP) of 125.5 mg/L (normal < 5 mg/L), prolonged activated partial thromboplastin time (aPTT) of 49 s (normal 25–35 s), international normalized ratio (INR) of 1.43 (normal 0.8–1.2), and severe thrombocytopenia with a platelet count of 30 × 10^9^/L (normal 150–400 × 10^9^/L). Anti-neutrophil cytoplasmic antibodies (ANCA) were negative for anti-PR3, while anti-MPO levels were borderline. Anti-Smith (anti-Sm) and antiphospholipid antibodies, including anticardiolipin (aCL), anti-β2-glycoprotein I (anti-β2GPI), and lupus anticoagulant, were negative, while complement levels (C3 and C4) were within normal limits. Antinuclear antibodies (ANA) were positive, with borderline reactivity against double-stranded DNA (dsDNA), without other clinical or laboratory features suggestive of systemic lupus erythematosus. A comprehensive thrombophilia work-up, including protein C and protein S levels, antithrombin activity, and factor V Leiden mutation, was unremarkable. Repeat CTA of the head, chest, and abdomen demonstrated no vascular pathology apart from hepatosplenomegaly.

**Figure 2:**
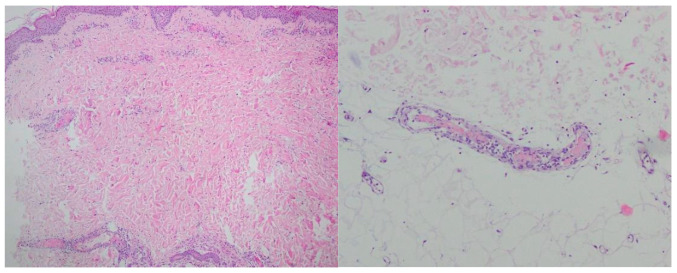
Histopathological examination. On the (**left**), showing vessels of the superficial and deep dermal plexus (H&E stain, original magnification ×4). On the (**right**), subcutaneous tissue with a small vessel occluded by a fibrin-rich thrombus. The vessel wall is largely preserved, with focal neutrophilic infiltration (H&E stain, ×200).

**Figure 3:**
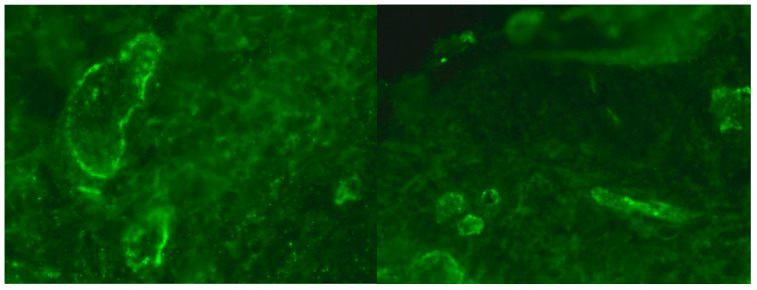
Direct immunofluorescence showing fine granular deposits of IgA (+) (**right**), IgG (±), and C3 (+) (**left**) in the walls of superficial dermal vessels.

A deep skin biopsy, including subcutaneous fat, was performed. Histopathological examination revealed widespread fibrin-rich thrombotic occlusion of vessels within the superficial and deep dermal plexus, with the most pronounced involvement of subcutaneous vessels. The vessel walls were largely preserved. Focal vessel wall necrosis with sparse neutrophilic infiltration was observed (Figure 2). Direct immunofluorescence demonstrated fine granular deposits of IgA (+), IgG1 (±), and C3 (+) in the walls of numerous superficial dermal blood vessels (Figure 3).

### 2.3. Treatment

Given the initial clinical suspicion of LCV, systemic therapy was initiated with intravenous methylprednisolone pulses (250 mg daily for 3 consecutive days), followed by oral corticosteroids with gradual tapering. Dapsone was additionally introduced at a dose of 50 mg daily and continued for one month, pending the histopathological diagnosis. In parallel, considering both the clinical features suggestive of a thrombotic component and the previously documented superficial vein thrombosis of the right upper limb on Doppler ultrasonography, anticoagulation with low-molecular-weight heparin (enoxaparin) was initiated from the beginning of hospitalization at a dose of 60 mg daily and constituted a key element of treatment. Topical therapy included betamethasone dipropionate combined with gentamicin. Pain management consisted of ketoprofen during hospitalization, followed by continuation of analgesic treatment with paracetamol. Anticoagulation was continued post-discharge with enoxaparin at a dose of 40 mg subcutaneously once daily for a total of 8 weeks.

### 2.4. Follow-Up and Outcomes

**Figure 4:**
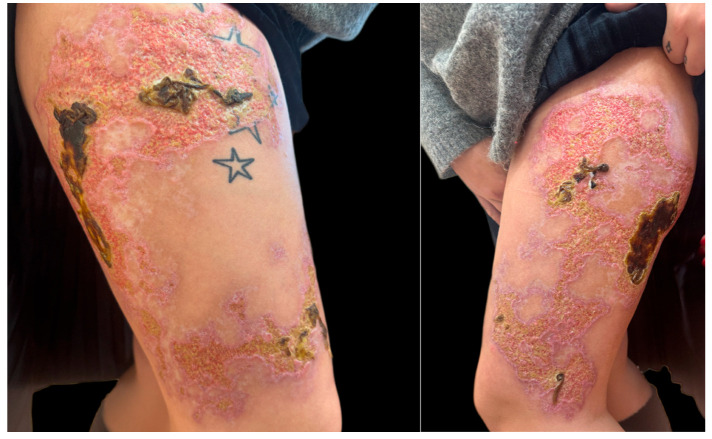
Clinical improvement after treatment. Marked regression of retiform purpura with partial re-epithelialization and residual post-inflammatory erythema and hyperpigmentation was observed eight weeks after therapy. Residual necrotic crusts are still present.

At 6 weeks after hospital discharge, a marked clinical improvement was observed, characterized by regression of skin lesions, absence of new purpuric foci, and significant reduction in pain (Figure 4). This early follow-up time point was selected to assess initial treatment response, while longer-term observation remains ongoing. Concomitantly, normalization of inflammatory markers, including C-reactive protein, as well as hematological parameters, particularly platelet count, was achieved. Coagulation parameters also returned to within normal limits. Based on the integration of clinical presentation, laboratory findings, and histopathological evaluation, a final diagnosis of STV was established. The patient remains under continuous multidisciplinary follow-up involving dermatology, vascular surgery, and psychiatry.

## 3. Discussion

### 3.1. Pathophysiology and Clinicopathologic Spectrum of STV

STV is a rare, non-inflammatory, occlusive disorder of the cutaneous microvasculature that can closely mimic inflammatory vasculitis, both clinically and histopathologically. In the literature, only a limited number of cases have been reported so far. Most reports describe STV in association with hypercoagulable states, chronic kidney disease, autoimmune disorders, or medications [1,2,3]. STV is characterized by extensive microvascular thrombosis predominantly affecting the subcutaneous tissue, with relative preservation of vessel wall architecture and minimal or absent inflammatory infiltrate [1,3]. Proposed mechanisms include acquired and inherited hypercoagulable states (such as antiphospholipid syndrome, protein C or protein S deficiency, factor V Leiden mutation, prothrombin G20210A mutation, and antithrombin deficiency), chronic kidney disease and end-stage renal disease, autoimmune and inflammatory disorders (including systemic lupus erythematosus, inflammatory bowel disease), severe infections and sepsis. Other proposed causes include vaccination, local tissue trauma or surgery, and exposure to prothrombotic medications, such as systemic glucocorticosteroids [1,3]. The presented case illustrates the diagnostic complexity of this spectrum. The classical definition of STV assumes the absence of vessel wall inflammation, fibrinoid necrosis, and significant immune deposits. In clinical and histopathological practice, this picture is not always clear. According to the literature, the histopathological appearance of purpuric lesions depends on lesion duration, and thrombotic and inflammatory features may overlap [4]. In later stages of thrombosis, features resembling leukocytoclastic vasculitis may be present, while advanced inflammatory lesions may also show occlusive vascular changes [4]. Therefore, biopsy findings should always be interpreted in the context of the clinical presentation and lesion duration. Similar overlap has been described in other thrombotic vasculopathies, including levamisole-associated vasculopathy and antiphospholipid syndrome-related skin lesions [5,6,7]. Because these changes often evolve over time and involve the deep dermis and subcutis, clinicopathologic correlation and adequately deep biopsy remain essential for accurate diagnosis [4,8]. In the presented case, thrombotic features were predominant, with fibrin thrombi present in vessels of the dermis and subcutaneous tissue. At the same time, focal vessel wall necrosis, mild neutrophilic infiltration with leukocytoclasia, and immune deposits on direct immunofluorescence were observed. This pattern does not fully correspond to a classical “pure” form of STV, but rather suggests STV with overlapping features of LCV.

### 3.2. Intravenous Injection of Crushed Tablets as a Trigger

An important aspect of this case is the time association between the development of cutaneous lesions and intravenous injection of crushed oral oxycodone and methylphenidate tablets. Oral pharmaceutical formulations commonly contain insoluble particles. These include microcrystalline cellulose, talc, and crospovidone, which are not intended for parenteral administration. When injected intravenously, these substances may act as foreign material capable of lodging within small-caliber vessels, leading to endothelial injury, platelet activation, and secondary thrombosis [9,10,11]. This mechanism has been well described in the pulmonary circulation, where intravenous injection of crushed tablets may result in pulmonary foreign-body embolization, granulomatous inflammation, and pulmonary hypertension [9,10,11]. It is conceivable that a similar process could affect the cutaneous and subcutaneous microvasculature and contribute to localized thrombotic vascular injury. In the present case, the diffuse thrombotic involvement of dermal and subcutaneous vessels, together with the history of intravenous injection of crushed oral medications, raises the possibility of particle-associated vascular injury, contributing to the observed clinicopathologic findings. However, we do not demonstrate the direct evidence supporting this mechanism, such as direct microscopic identification. Although cutaneous complications of intravenous injection of crushed tablets, including ischemic necrosis, soft-tissue infections, compartment syndrome, and limb ischemia, have previously been reported, clinicopathologic presentations resembling STV appear to be rarely described [12,13]. Therefore, this case should be interpreted cautiously as a possible observation expanding the spectrum of thrombotic and thrombotic-inflammatory cutaneous vasculopathies associated with intravenous drug use.

### 3.3. Differential Diagnosis of STV

Clinically, STV typically presents with retiform purpura, livedo racemosa, painful subcutaneous nodules, and rapidly progressive necrosis, predominantly affecting areas rich in subcutaneous fat [1,3,14]. The differential diagnosis is broad and includes calciphylaxis, LCV, disseminated intravascular coagulation, systemic thrombotic microangiopathies, embolic vasculopathies, and antiphospholipid syndrome [1].

Calciphylaxis is one of the most important differential diagnoses because it may present with painful livedoid or retiform purpura that progresses to necrosis, often also involving adipose-rich areas [2,15]. Histopathologically, it is characterized by vascular calcification, intimal proliferation, and thrombosis [16]. In the present case, the absence of vascular calcification on routine histology and lack of clinical features of chronic kidney disease argued against calciphylaxis, although an early or non-calcified form cannot be fully excluded.

LCV was considered in the differential diagnosis because of focal leukocytoclasia, vessel wall damage, and positive direct immunofluorescence findings [17]. At the same time, fibrin-rich thrombi involving dermal and subcutaneous vessels supported STV. Clinically, the presentation was dominated by rapidly progressive retiform purpura and necrotic lesions rather than palpable purpura typical of classic LCV. Taken together, these findings suggested STV with LCV features rather than isolated small-vessel vasculitis.

Disseminated intravascular coagulation and systemic thrombotic microangiopathies were considered because of fever, thrombocytopenia, elevated inflammatory markers, and coagulation abnormalities [18,19,20]. However, the absence of hypofibrinogenemia, microangiopathic hemolysis, organ dysfunction, severe coagulopathy, or progressive septic course made these diagnoses less likely. Nevertheless, systemic coagulopathy could not be completely excluded.

Embolic vasculopathy was also considered; however, no material or proximal embolic source was identified histopathologically or clinically.

Antiphospholipid syndrome and lupus-associated vasculopathy were also considered in view of positive antinuclear antibodies and borderline anti-dsDNA reactivity. However, antiphospholipid antibodies were negative and no additional clinical or laboratory findings consistent with systemic lupus erythematosus were present.

Comprehensive differential characteristics of STV are summarized in Table 1.

### 3.4. Therapeutic Implications

Therapeutic management in this case included anticoagulation, systemic corticosteroids, and dapsone. While anticoagulation addresses the primary thrombotic process, immunomodulatory therapy was guided by overlapping vasculitic features, including leukocytoclasia and immune deposits on direct immunofluorescence. This combined approach resulted in rapid clinical improvement, supporting the concept of a mixed thrombotic-inflammatory process rather than a purely occlusive disorder.

Due to the rarity of STV, there are currently no standardized treatment guidelines, and therapeutic strategies are largely based on individual case reports and small case series. Management is therefore typically tailored to the presumed underlying mechanism and clinical presentation, including the use of anticoagulation, immunosuppressive or anti-inflammatory agents, and elimination of potential triggering factors.

### 3.5. Limitations

This article has several limitations that should be acknowledged. First, the history of intravenous substance use was based on patient self-report and may have been incomplete or imprecise, particularly regarding the exact timing, frequency, and composition of injected substances. No toxicological confirmation was available. Second, although intravenous injection of crushed oral oxycodone and methylphenidate tablets was considered a potential trigger, the proposed mechanism of particle-induced microvascular occlusion remains hypothetical. No foreign particulate material was identified histopathologically, and additional techniques such as polarized light microscopy were not performed. Therefore, a direct causal relationship between injected tablet material and the observed vascular pathology cannot be definitively established. Thus, some degree of diagnostic uncertainty persists. Fourth, despite extensive laboratory and imaging evaluation, systemic coagulopathies and thrombotic microangiopathic processes cannot be completely excluded, although the overall clinical course and additional investigations argued against disseminated intravascular coagulation or systemic thrombotic microangiopathy as the primary diagnosis. Finally, histochemical staining for vascular calcification, such as von Kossa or Alizarin red staining, was not performed. Therefore, an early or non-calcified form of calciphylaxis cannot be definitively excluded. In addition, only a single biopsy specimen was obtained, limiting assessment of temporal histopathologic evolution of the lesions.

## 4. Conclusions

In conclusion, this case highlights the importance of considering thrombotic vasculopathy in the differential diagnosis of retiform purpura and necrotic skin lesions, particularly in patients with a history of intravenous drug use. The presented findings suggest STV with overlapping features of LCV, emphasizing the potential coexistence of thrombotic and inflammatory vasculitic changes. This case may also suggest a possible role of intravenously injected particulate matter from oral medications as a trigger. Recognition of this mechanism and its clinicopathologic overlap is essential for accurate diagnosis and appropriate management, and further studies are needed to better define the spectrum and pathogenesis of particle-induced cutaneous vasculopathies.

## Figures and Tables

**Table 1:** Differential diagnosis of thrombotic and inflammatory cutaneous vasculopathies (based on [1,2,3,4,8,14,15,16,17,18,19,20,21,22,23,24,25].

Feature	Subcutaneous Thrombotic Vasculopathy (STV)	Calciphylaxis	Inflammatory Vasculitis	Leukocytoclastic Vasculitis (LCV)	Disseminated Intravascular Coagulation (DIC)	Purpura Fulminans	Embolic Vasculopathy
**Fibrinoid necrosis**	Absent	Absent	Common	Common	Usually absent	May be observed	Absent
**Immune complex deposition (direct immunofluorescence)**	Negative or minimal; may show nonspecific deposits in secondary inflammatory changes	Negative	Frequently positive (IgG, IgA, C3)	Positive (IgA/IgG/IgM and C3 deposition in vessel walls)	Negative or nonspecific	Negative or nonspecific	Negative
**Inflammation of the vessel wall**	Absent or minimal, secondary	Minimal or absent	Prominent, destructive	Present neutrophilic vasculitis	Minimal or absent	Minimal or absent	Absent
**Predominant vessel involvement**	Small-caliber vessels, predominantly subcutaneous microvasculature	Small vessels of the subcutaneous adipose tissue	Small and medium-sized vessels of the dermis ± subcutis	Small vessels of the superficial and mid-dermis, especially postcapillary venules	Widespread small-vessel and microvascular thrombosis in the skin and internal organs	Small dermal vessels, capillaries, and venules with widespread thrombosis	Variable: arteries, arterioles, or microvasculature
**Vascular calcification**	Absent	Present	Absent	Absent	Absent	Absent	Absent
**Primary pathogenetic mechanism**	Non-inflammatory microvascular thrombosis	Medial vascular calcification with superimposed thrombosis and ischemia	Immune-mediated inflammation and destruction of the vessel wall	Immune complex-mediated small vessel vasculitis	Systemic coagulation activation with consumption of platelets and clotting factors, resulting in microvascular thrombosis and bleeding	Thrombotic vasculopathy due to dysregulated coagulation	Mechanical occlusion by embolic material (cholesterol, thrombus, foreign particles, septic emboli)
**Typical clinical presentation**	Retiform purpura, painful subcutaneous nodules, rapidly progressive necrosis	Severe pain, livedo racemosa, necrotic ulcers, high risk of infection	Palpable purpura, ulcers, nodules; often systemic symptoms	Palpable purpura, usually on lower extremities; may include petechiae, urticarial lesions, vesicles/bullae, ulcers, burning, or pain. Systemic symptoms depend on cause	Petechiae, purpura, ecchymoses, hemorrhagic bullae, retiform purpura, acral ischemia, skin necrosis, bleeding, and signs of systemic illness	Retiform purpura, rapidly progressive skin necrosis, hemorrhagic bullae, and systemic coagulopathy (DIC)	Livedo racemosa, acral ischemia, digital infarcts, tissue necrosis
**Main therapeutic strategy**	Anticoagulation (unfractionated or low-molecular-weight heparin), removal of triggering factors, supportive wound care	Multimodal therapy: sodium thiosulfate, discontinuation of warfarin, intensive wound care, pain control, management of calcium–phosphate balance, treatment of infection	Immunosuppression (systemic corticosteroids, cyclophosphamide, rituximab, azathioprine, methotrexate, mycophenolate mofetil)	Trigger removal, supportive care, systemic corticosteroids and immunosuppressants (azathioprine, methotrexate, mycophenolate mofetil, cyclophosphamide, rituximab)	Treatment of the underlying cause, supportive care, blood product replacement when bleeding or high bleeding risk is present, and anticoagulation in selected thrombotic cases	Management of underlying cause (e.g., antibiotics for sepsis), anticoagulation (heparin), replacement therapy (protein C), intensive supportive care (wound care, hemodynamic support)	Treatment of embolic source (antibiotics for infective endocarditis, anticoagulation, antiplatelet therapy, vascular or cardiac intervention)

## Data Availability

The data presented in this study are available on request from the corresponding author.
